# Analyzing Mega City-Regions through Integrating Urbanization and Eco-Environment Systems: A Case Study of the Beijing-Tianjin-Hebei Region

**DOI:** 10.3390/ijerph16010114

**Published:** 2019-01-03

**Authors:** Li Tian, Gaofeng Xu, Chenjing Fan, Yue Zhang, Chaolin Gu, Yang Zhang

**Affiliations:** Department of Urban Planning, Tsinghua University, Beijing 100084, China; xugf16@mails.tsinghua.edu.cn (G.X.); fanchenj@mail.tsinghua.edu.cn (C.F.); gucl@mail.tsinghua.edu.cn (C.G.); zyang17@mails.tsinghua.edu.cn (Y.Z.)

**Keywords:** mega city-region, urbanization, eco-environment, system dynamic model, land contamination

## Abstract

The high-speed economic growth of mega city-regions in China has been characterized by rapid urbanization accompanied by a series of environmental issues ranging from widespread soil contamination to groundwater depletion. This article begins with an analysis of the interaction between urbanization and the ecological system and reviews existing frameworks for analyzing urban and ecological systems. By taking the Beijing-Tianjin-Hebei region as an example, the article introduces a conceptual framework to analyze mega city-regions and forecast possible interactions between urbanization and eco-environment by applying simulation model. The proposed framework and its components can provide guidance to identify the impacts of urbanization and external forces such as globalization on eco-environment by integrating the internal and external factors, synthesize the complex components of mega city-regions in databases, understand and diagnose the casual relationship between urban policies and ecological consequences.

## 1. Introduction

At the beginning of the twenty-first century, a new type of urban form, the mega city-region, first anticipated by Gottmann [1] emerged across the globe [2]. As globalization intensifies, these city-regions come to pose many challenging problems to policy-makers and researchers. Urban land, including both soil and groundwater, is often contaminated due to historical industrial operations, heavy traffic activities, as well as urban runoff carrying a variety of anthropogenic pollutants [3,4,5]. The mega city-region is a complex system which involves social, economic, political and ecological sub-systems, and every sub-system forms an inalienable organism with internal interconnection and mutual restriction as well as external inflows. The development of a mega city-region will be affected if any component in it changes. For instance, increased population will exert a huge impact on land and water resources. Due to large population density and large-scale human activities, the ecosystem is fragile and unstable. Compared with natural ecosystem, the resource utilization efficiency is much lower in urban system, and material recycling is linear instead of being close. A great amount of material and energy is exported into the environment in the form of waste, resulting in extensive environmental pollution [6,7].

As one of the major mega city-regions in China, the Beijing-Tianjin-Hebei (BTH) region has experienced rapid urbanization and industrialization since the reform opening in 1978. It has long been sensitive to profound conflict between socioeconomic development and environmental pollution in the process of achieving sustainable development. From 1980 to 2015, the urbanization rate increased from 38.86% to 62.5%, and GDP increased from 458.7 to 68,905.2 billion Yuan [8]. Accompanying rapid economic growth was a serious water shortage and air pollution and both of these aroused nationwide concerns.

Su et al. [9] argue that the mega-region policy achieves the economic goal, but fails to achieve the goals of environmental protection, and social equity in China. In order to understand the impacts of urbanization on eco-environment, developing a conceptual framework has become increasingly important. Understanding how social and ecological systems interact with each other is essential for sustainable development. Over the last several decades, theories such as social-ecological systems (SES), urban metabolism, ecological-economics (EE) and models such as system dynamics model (SD) which analyze complex urban and ecological systems have emerged to help us understand the mechanism between urban development and eco-environment system, and to simulate the environmental consequences by different policy, which will help in prioritizing the policies. There has been a wealth of empirical research that apply these theories/models [10,11,12,13,14,15,16]. Nevertheless, in rapidly urbanizing regions, how to establish an integrated model which addresses the interaction among the components of urbanization and eco-environment has been fairly scarce due to the complexity of interactions among various system components, and the external influence under the backdrop of globalization makes the situation more complicated.

In this research, we propose a framework of a regional sustainable development assessment method which integrate interaction among external inflows, internal urban system and eco-environment, taking the BTH region as an example. The significance of this conceptual framework lies in that it can provide guidance to identify the impacts of external forces and urban development on eco-environment by integrating the internal and external factors. Moreover, a systematic scientific platform for quantitative research of urbanization and eco-environment interaction can be established for simulation and multi-scene analysis in policy-making. The characteristics of this framework is that it addresses the complex dynamics of urbanization and eco-environment in rapid developing regions, avoiding oversimplifying either the ecological or the urban domain, and focuses on interactions that drive the development of the coupled system over time. This paper is organized as follows: firstly, it examines the interaction between urbanization and the eco-environment system and reviews existing frameworks for analyzing urban and eco-environment systems. Moreover, it summarizes the frequently used indicators in urban and ecological systems. In the following section, taking the BTH region as a case, we design a conceptual framework which identifies the challenges facing this region, divides the urbanization and eco-environment systems into six sub-systems and analyzes the correlations between the six sub-systems and external inflow at the cross-scales: global, regional and city. It then proposes the simulation models to examine the interrelations among the internal subsystems, and synthesizes the complex components of mega city-regions in databases to predict the potential impact of globalization, mega project, national policies and urban development on eco-environment.

### 1.1. Urbanization, Globalization and Their Impacts on Eco-Environment

Over the past century, the world’s urban population increased from about 200 million in 1900 to 4.22 billion in 2018. In 2008, the global urban population exceeded the rural population and over 56.8 percent of people across the globe were living in urban areas in 2018 [17].

Urbanization creates many human-dominated landscapes and 24% of earth’s terrestrial experienced decline in ecosystem function and productivity, due to the transformation of land for human activities [18,19]. Over the past one hundred years, ecosystem processes changed more rapidly than at any time in the human history [20]. The literature of ecological and environmental science before the 1990s viewed the urbanization process merely as environmental ills that caused degradation of ecosystem services. The first negative we see in urbanization is how it affects biodiversity [21]. Over the past century, the species extinct at the speed of 1000 times of historical rate. Secondly, urbanization has increased the concentration of pollution and changed the climate condition. The atmospheric concentration of carbon dioxide has increased by 32% since 1750, approximately 60% of that increase has taken place since 1959, primarily due to the combustion of production activities and land use changes during the global high-speed urbanization stage [20,22]. Thirdly, urbanization has caused more serious natural disaster losses. The frequency and impact of floods and fires has increased dramatically over the past half century. Last but not least, urbanization changes hydrologic systems, making them more unstable [23,24]. Urban construction significantly increases impervious area, and the impact seen includes much more flooding and peak flow volumes, increased sediment loadings, loss of aquatic/riparian habitat, changes in stream physical characteristics, decreased base flow and increased stream temperature [25], environmental pollution from a river had caused the contamination of soil, groundwater, and air as well [26,27]. Although urbanization has had a negative impact on the ecological system, some findings since 2000 suggest that urbanization is not always associated with environmental degradation. Bettencourt [4] argues that with economic development, urbanization raises environmental awareness, protection and quality. In large urban agglomerations, solutions for sustainable development are more accessible through new technological tools and novel institutional arrangements. Empirical research indicates that social factors such as economic stimulus, population control, governance and regulation in the urbanization process may contribute to an improved environment [28,29,30,31]. In developed areas, governments encourage producers to clean their own waste or seek alternative production processes to reduce emissions [20], in developing countries, there is already an intention to actively repair environmental pollution [32].This complex relationship between environment and economic development is called the environmental Kuznets curve (EKC) hypothesis: environmental performance tends to get worse as modern economy grows until average income reaches a certain point [29]. Although there is continued debate on Kuznets curve, evidence confirms that some environmental health indicators, such as water and air pollution, manifests an inverted U-shaped curve [33].

Since the 21st century, globalization has started to exert dramatic impacts on urbanization and ecosystem at a global scale. The impact of globalization on mega city-regions has been two-fold, resulting in positives and negatives. On the one hand, globalization has promoted the rational allocation of markets and the large-scale transfer of rural labour force to urban areas, and speeded up the urbanization process. Moreover, globalization has promoted global environmental governance and has helped extend the idea of sustainable development and the circular economy [34]. Moreover, globalization has brought about the sharing of innovative technologies and management tools to provide solutions for governance in developing countries [35]. On the other hand, globalization attaches the blindness of capitalist production and the global expansion of capital [36], which directly brings about deterioration in human development. In general, the unbalanced development of the global economy has aggravated the deterioration of the ecological environment [37].

Generally speaking, a consensus on the relationship among urbanization, globalization and eco-environment system has not been achieved, varying from country to country, from region to region. The integrated quantitative measurement of this link has been proved to be a very difficult task.

### 1.2. Existing Frameworks of Analyzing Urbanization and Eco-Environment Systems

Over the last several decades, the complexity of interactions between urbanization and eco-environment has attracted wide concern and research from various perspectives have emerged (Table 1). They can be summarized as the following three types according to different focuses and perspectives. 

### 1.3. The City as a Special Type of Ecosystem and Its Demand for Ecological Services

From the perspective of an ecosystem, urban ecological studies focus on biodiversity, urban metabolism, and ecosystem services ranging from small towns to major metropolitan regions [44] and they view the urban ecological system as a part of the entire ecosystem and emphasize the impact of urban development on the ecological system. However, the interaction between the urban and ecological system is not their focus.

“Urban metabolism” is a typical model which views the city as an ecological system and is defined as ‘the sum total interaction of the technical and socio-economic processes that occur in cities, resulting in growth, production of energy, and elimination of waste’ [45]. It includes production, transformation, consumption and exchange of materials, resources, energy and services between a city and its external environment. The concept of an urban metabolic process is frequently used to measure urban or regional socio-economic metabolism [16], and it provides a holistic perspective to encompass all activities in a city. The analysis of urban metabolic processes in China reveals imbalance between urban and ecological system, such as resource and energy shortages, eco-environmental degradation, falling quality of life in the course of urban and regional development [6]. The focuses of urban metabolism research are the stocks and flows of different kinds of ecosystem, energy, or biophysical elements; however, they do not discuss people-oriented goals [46].

MEA [20] addresses people-oriented goals while analyzing the function of the ecological system and defines ecosystem services as ‘the benefits people obtain from ecosystems,’ including (1) ‘provisioning services’ such as food and water; (2) ‘regulating services’, for example, standard of air and water, regulation of climate, floods, diseases, hazards, and noise); (3) ‘cultural services’, including recreational, spiritual, religious benefits; and (4) ‘supporting services’ such as soil formation, primary production, and nutrient cycling. They are mainly concerned with urban demand as an ecosystem, focusing on the impact of urbanization on ecosystem services [47].

### 1.4. Frameworks for Analyzing the Complex and Coupled Urban and Ecological System

Recently, more and more scholars have realized the coupled relationship between urban and ecological systems. They have come to adopt transdisciplinary and interdisciplinary approaches to analyze interactions and mechanisms of the coupled systems. Representative theoretical frameworks include ecological-economics, socio-ecological system and human-environment systems.

### 1.5. Ecological-Economics (EE)

In the 1980s, ecological economics emerged as a modern discipline, and it focuses on the interdependence and coevolution of economy growth and natural ecosystems at temporal and spatial dimensions [48]. Faber [49] defines the focus of ecological economics as nature, justice, and time. While analyzing and evaluating ecological economics, intergenerational equity, irreversibility of environmental change, and uncertainty of long-term outcomes are usually used as criteria. Different from mainstream economic approaches such as cost-benefit analysis, ecological economist Costanza [13] proposes four basic types of capital assets: (1) built or manufactured capital; (2) human capital; (3) social capital; and (4) natural capital. He argues that the scale and distribution of the urban component should be determined by social decisions reflecting ecological limits, the distribution and resource allocation system must sufficiently recognize the value of social and natural capital. While the analytical framework of EE has become more popular, there are also critiques which regard EE as being unable to address the underlying problems with mainstream economics [50].

### 1.6. Socio-Ecological System (SES)

Until the past few decades, the interaction between social and natural sciences was scarce in dealing with social and ecological systems. Since the 1960s the concept of socio-ecological systems has been used to emphasize the integration of human and nature, and SES argues that social and ecological systems are linked through feedback mechanisms, which display both resilience and complexity [51]. Ostrom [52] initially put forward a multilevel, nested framework to analyze the SES, which contains four core subsystems: resource systems, resource units, governance systems, and users. Recently, many research studies have applied the SES framework in the integration of resource allocation, environment, pollution, users, social organizations and government regulation [53]. Initiated from the coupling relationship between climate change and human activities, much research focuses on incorporating human and natural components simultaneously [54], as well as human-nature interactions and population-resource system management [55]. While SES is a fast-growing interdisciplinary field, it is complex and adaptive and requires continuous testing, learning, knowledge development and understanding while dealing with change and uncertainty [56].

### 1.7. Driving Forces-Pressures-States-Impacts-Responses (DPSIR)

The DPSIR framework started in the late 1970s. DPSIR provides a framework for describing the interactions between humans and the environment, and it is a tool that integrates the environmental and socio-economic impacts for detail analysis. According to the DPSIR framework, social and economic developments and natural conditions exert pressure on the environment, leading to the changes of the environmental state. DPSIR is meaningful for policy design in order to better understand the issues of environment at multiple levels and spatial scales, and it establishes indicators of environment in relation to human activities. Nevertheless, DPSIR also suffers from some drawbacks such as simplicity and linearity which can weaken the reliability of analysis [41].

### 1.8. City as a Nexus System under Governance

Looking inside the urban system, we find that governance and innovation may exert significant impact upon the urban and ecological system. Jacobs [42] incorporates ideas from different theories, such as world/global city, urban growth regime, agglomeration, and nested city theories, and puts forward the city nexus concept. He argues that a combination of politico-governmental (State), market-economic (Market), civil-societal (Societal), and geographic-natural (Geo-spatial) activities have jointly shaped economic and spatial outcomes in mega city-regions. Comprehensive policy could lead to sustainable urban development and enhance financial benefits by creating favorable conditions. The constant flow of new capital investment, firms, and people generally lead to political fragmentation, income inequality, and geographic dispersion [42,57]. Lizarralde et al. [58] also highlighted the importance of policy-making agendas in enhancing urban planning sustainability and resilience.

Moreover, cities are considered the centers of innovation and can advance sustainable management and spatial development strategies [59]. Rotmans et al. [60] address the necessity to develop an integrated governance system that can capture the nexus of the environmental, socio-cultural and economic factors that shape the development of creative and sustainable cities. Smart systems can reduce vulnerability, and it is imperative for cities to be resilient if they can be considered smart. Resilience shares much with other key contemporary urban goals such as sustainability, governance and economic development [30,31,61]. Additionally, Kattel et al. [14] propose an Ecology-Environment and Human Health-Urban design management framework for effective urban planning and social harmony in order to better understand the complementary roles of ecological system in urban development and the functioning of ecosystems and ecological resilience in a complex human-dominated landscape.

**Figure 1:**
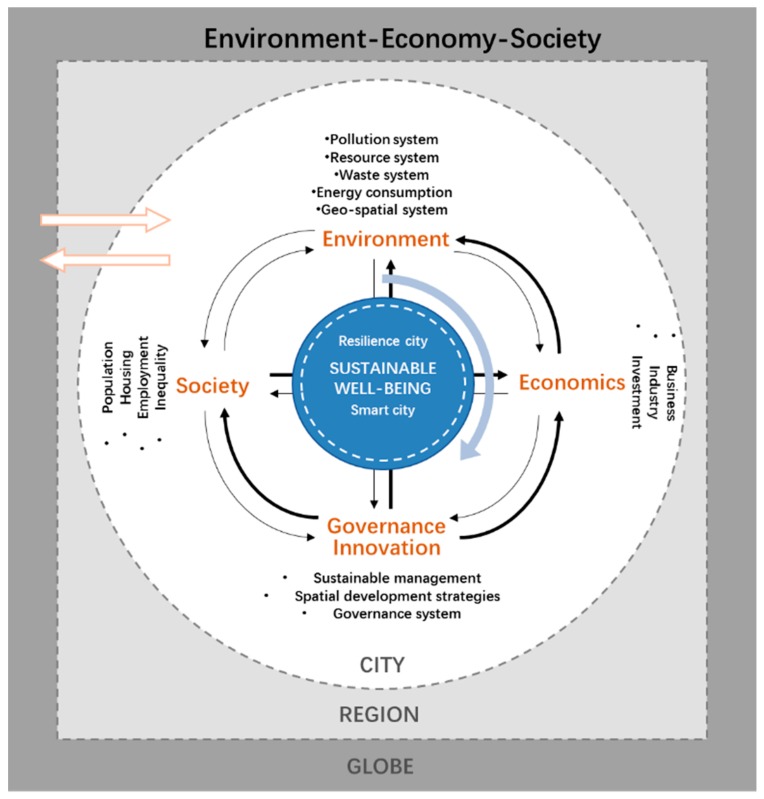
Complex urbanization and eco-environment system at cross-scales. Source: Drawn by authors.

In general, while analyzing the complex and coupled urban and ecological systems, scholars from different fields have put forward various models/theoretical frameworks to explore the impact of urban development on the ecological system and their interactions (Figure 1). Based on these frameworks, there has been rich empirical research conducted in developed countries or city-regions.

**Figure 2:**
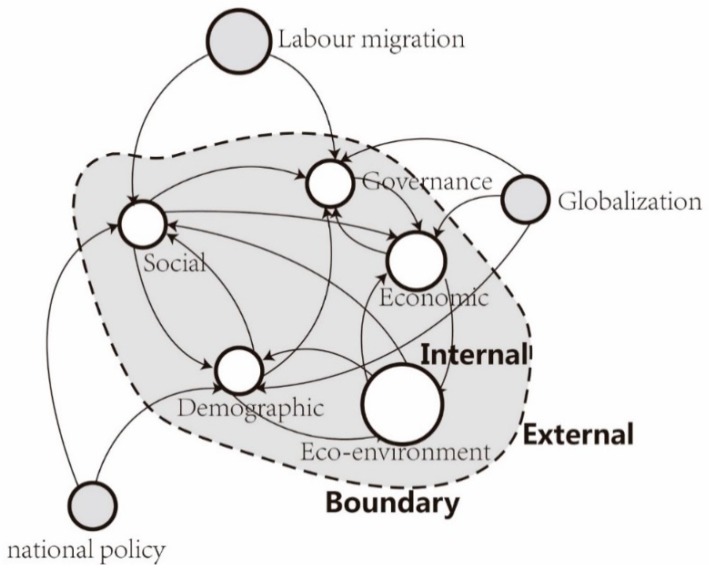
Illustration of external and internal influence on eco-environment in mega city-region. Source: Drawn by authors.

Nevertheless, an integrated and holistic research on the relationship between urbanization and eco-environment in rapid urbanizing regions is fairly scarce, and this is attributed to the dynamic and complex nature of these areas. In reality, there have been external and internal factors which exert dramatic influences on the development of these regions, and the contradiction between urban and ecological systems is more prominent and the demand for system coordination is more pressing compared with developed regions. These regions are more likely to be affected by the external environment, such as globalization, labour migration and national investment (Figure 2). Meanwhile, the internal interaction among demographic, social, economic, and governance can exert influence on eco-environment to a large extent. Considering the variety and characteristics of rapidly growing regions, it is imperative to develop a conceptual framework suitable for evaluating, analyzing, and predicting potential interactions between urbanization and eco-environment systems from both internal and external perspectives.

## 2. Indicators Applied in Analysis of Integrated Urbanization and Eco-Environment Systems

The selection of indicators plays an essential role in developing the framework of urbanization and eco-environment systems. In order to understand which indicators have been frequently used in the systems, we conducted extensive literature review on existing research; moreover, we examine the indicators developed by international organizations such as the UN and the World Bank. We then categorized these indicators into ten major categories in a combined database to identify the most frequently used indicators. Based on relevant literature, we select indicators based upon the following criteria:

(1) High-level relevance with urban-ecology, sustainability, urbanization and eco-cities;
(2) High-level relevance with mechanism or driving force of urbanization and eco-environment;
(3) Evaluations conducted at the metropolitan/regional level;
(4) Clarity of indicator definitions;
(5) Clarity of indicator selection criteria and methodology.

Next, we went through international and local indicator systems. In 2015, The United Nations approved the Sustainable Development Goals (SDG) in the UN 2030 Agenda for Sustainable Development. With 17 goals, 169 targets, and over 200 indicators, the SDG covers three dimensions of sustainable development: social, economic, and environmental dimensions and their institutional/governance aspects, and addresses some systemic barriers of sustainable development. Likewise, in the 2017 edition of the World Development Indicators [62] proposed by the World Bank, cross-country comparable data was compiled. The database covers six main sections (world view, poverty and prosperity, people, environment, economy, state and market, global links) and contains more than 1400 time series indicators for 217 economies and more than 40 country groups. The International Organization for Standardization proposed ISO-37120 “Sustainable Development of Communities Indicators for City Services and Quality of Life”, which considers telecommunication and innovation, transportation and urban planning as key factors in sustainable development along with the others mentioned above. In 2014, the Japanese government put forward the “Future City” model for sustainable cities with superior environmental technologies, core infrastructure and resilience [63]. Moreover, some indicators have been proposed for specific habitats and environments. Upon reviewing international standards, the following categories of the indicator systems can be identified:

- Indicator systems that comprehensively measure social, ecological, economic, political, and cultural systems and are internationally applicable: SDG [17], WDI [62].
- Indicator systems that comprehensively measure social-ecological systems and are nationally or locally applicable: Japan Future City Initiatives 2011, etc. [63].
- Indicator systems focused on a specific habitat or environment in the urban context: urban forest ESG indicator [64], urban landscape, etc.
- Indicator systems focused on relationship or flows between economy and ecology
- Indicator systems focused on a certain function of economy to society: natural services.

**Figure 3:**
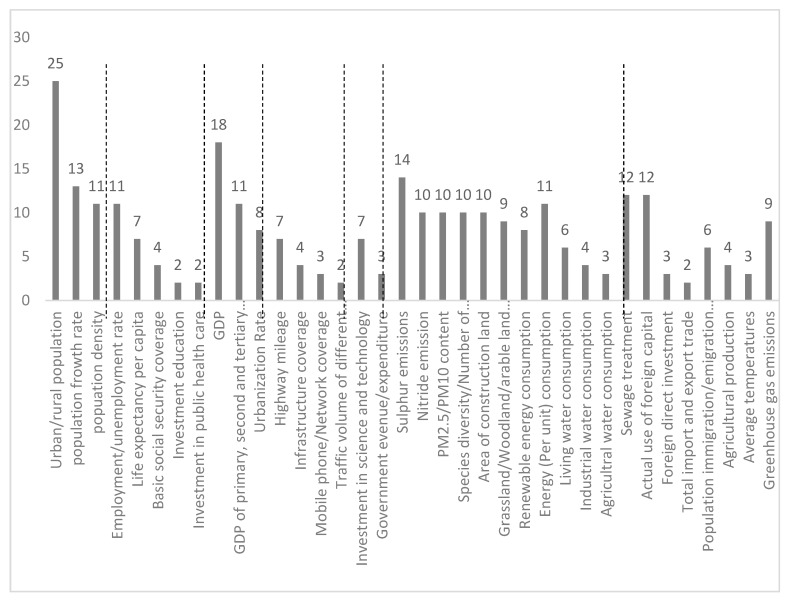
Frequency of indicators in literature and international standards Source: Drawn by authors.

These indicators are mostly rooted in the global and national level, and stem from various purposes. It is necessary to screen the relevant systems for regional and urban development. Based on the results of literature and international standards review, we record the frequencies of indicators (Figure 3). After summarizing the frequently used indicators, we divide urbanization system into six categories: demographic, society, economy, infrastructure, governance and innovation, and eco-environment, which is classified into water, territorial, air, energy/resources and creature.

## 3. A Conceptual Framework for Analyzing Urbanization and Eco-Environment Systems in Mega-City Region: An Example of the Beijing-Tianjin-Hebei Region

With reference to the above-mentioned framework, we diagnose the problems and challenges facing mega city-regions of China and propose a five-step conceptual framework to guide the analysis of complex and coupled urbanization and eco-environment systems in mega city-regions. Since this conceptual framework is problem and objectives-oriented, the first step is to identify the major place-specific problems and challenges to be diagnosed. This step should involve relevant stakeholders, in particular, various levels of governments, institutions, firms, and local residents in the Chinese context, which can help to diagnose problems precisely and initiate a process of co-design efforts [65]. The second step is to put the mega city-region into a global context and analyze the potential positive/negative impacts of globalization on the urbanization and eco-environment system of mega city-regions. Step 3 devolves the urbanization and eco-environment system of mega city-regions into six sub-systems: demographic, society, economic, environmental, governance, and transport systems, and their inter-dependency is also analyzed. Based on data availability, Step 4 further defines the variables in each sub-system. With the input of these variables, Step 5 suggests a simulation model to examine the impacts of urbanization on the eco-environment system.

### 3.1. Step 1: Diagnose Problems and Challenges Facing Mega City-Regions

**Figure 4:**
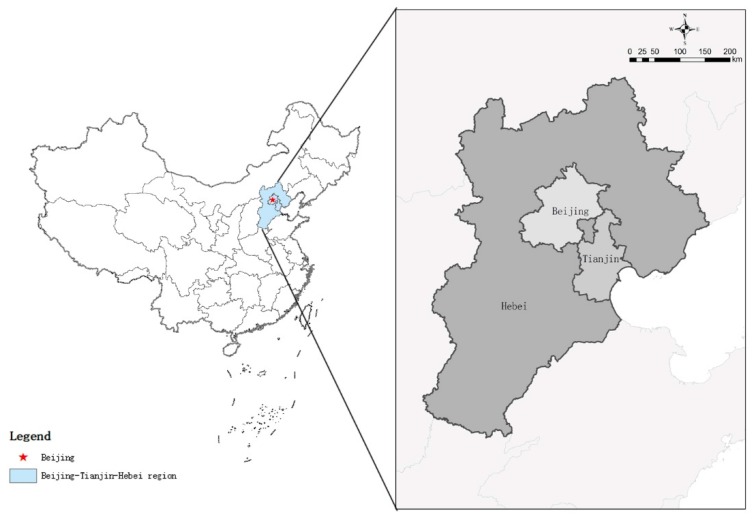
Location of the BTH region in China. Source: Drawn by authors. (BHT: Beijing-Tianjin-Hebei.).

Located in central China (Figure 4), the BTH region is one of the three most developed urban agglomerations in the country. It covers a land area of 218,000 km^2^ and the population reached 110 million in 2014. Since the opening reform, the BTH region has experienced rapid urbanization and industrialization. In 2015, the BTH region created 10.2% of the national GDP with its 2.25% of the national land area and 8.1% of the national population [66]. Meanwhile, 7.72% of waste water, 9.35% of waste gas emission and 14.74% of industrial solid waste came from the BTH region in 2012 [67]. This region has long faced increasingly serious resource and environmental problems, in particular, air pollution and water resource shortage and an enlarging regional disparity in social-economic development.

The annual water supply of the BTH region was 2.78 billion m^3^ in 2014, and the water resource per capita is only 1/9 of the national average and around 70% of the water came from underground exploitation [68]. Chen et al. [69] find that land subsided with a rate greater than 100 mm/year in the eastern part of Beijing from 2003 to 2011.The air quality of the BTH region has long been a worldwide concern. In 2015, the days of severe pollution reached 154. According to research of the Chinese Academy of Sciences, at least 25% of industrial pollution is contributed to smog in Beijing and the source of air pollution in Beijing was mostly from the outside surrounding provinces, especially Hebei, where the manufacturing industry such as steel production takes a dominant share of the economy [67]. Limited land resources is another factor constraining the development of the BTH region. From 2004 to 2013, the built-up area increased 10.5% in Beijing, 49.4% in Tianjin, and 43.1% in Hebei [54], leading to a significant loss of farmland. 

Regional disparity has long been a barrier for sustainable development in the BTH region. Compared with Beijing and Tianjin, Hebei is considerably disadvantaged in social and economic development (Table 2). In 2015, the urbanization rate of Beijing and Tianjin reached above 80% but that of Hebei was only 51.33% in 2015. The GDP per capita of both Beijing and Tianjin exceeded 100,000 yuan in 2015, while that of Hebei was only 40% of that in Beijing and Tianjin. Likewise, the disposable incomes of households in Beijing and Tianjin were much higher than that of Hebei. In terms of educational and medical services, Beijing stands out in the BTH region. Compared with Beijing and Tianjin, the secondary industry took a dominant share in the industrial structure of Hebei, among which the iron and steel industry was the pillar industry. Hebei has long been the top iron and steel producer in China, accounting for 23% of all iron and steel produced in the country [70], and it has been regarded as one of major reasons for heavy smog in the BTH region. In general, rapid urbanization and industrialization have made the ecological environment of BTH increasingly fragile. Rapid population growth and reliance on the manufacturing industry have been two key factors for threatening the conservation of the environment [54,67].

### 3.2. Step 2: Defining the External Inflow into Mega City-Regions

As above-mentioned, the globalization has significant influences on society, environment, government governance and innovation, and it reconfigures the economic structure, social structure and spatial layout of key node cities in the world. Positive effects of globalization on the BTH Region are revealed at the following four aspects: (1) Import trade and export remarkably raises the urbanization level [71]; (2) The global market links facilitate the upgrading of the service industry (especially finance, logistics and productive service industry) and promotes the formation of the BTH Free Trade Zone, culture and creativity parks and other special legal, social and cultural spaces, such as the Tianjin Free Trade Zone and the 798 Arts Zone; (3) Industrial upgrading caused by the information industry redistributes the spatial layout of population and a headquarters economy gives impetus to talent gathering while industrial suburbanization helps population decentralization become a reality; (4) Spatial integration of BTH region is promoted in terms of population distribution, regional traffic, portal functions, ecological environment and other aspects [72].

The negative environmental effects of globalization are also seen in the BTH region. According to the research of Zhao [73] on Foreign Direct Investment (FDI) and environmental quality in the BTH region from 1995 to 2013, depending on the industry types, the FDI in Beijing reduced regional environmental pollution, the FDI in Tianjin aggravated environmental pollution, while the FDI in Hebei had no obvious impact on environmental quality. With the upgrading of the industrial structure, the interaction between urbanization and the eco-environment in the BTH region begins to follow the Kuznets Curve [74].

Under the backdrop of globalization, the external influences of mega city-regions are diversified. Some researches suggests that labour mobility, market environment and foreign investments are related to the globalization [75] Global cities need a variety of cultural and recreational facilities for social networks, which greatly promote the large shopping center, tourism, sports facilities and social infrastructure to support the consumer-oriented mega-project. Mega projects often become engines of urban or regional development and promote the formation of new industrial space, urban development and spatial restructuring [76], attracting a large amount of labor migration. In China, national policy has significant impacts on urbanization and eco-environment of mega city-regions. On the one hand, the national policy changes overall market environment, which has important influence on the investment outside the region. On the other hand, national policies such as household registration policy, employment policy and mid-term and long-term national strategy dramatically affect migration, investment direction [77] and human health [78]. At the same time, global climate change as an external factor, such as carbon dioxide agglomeration and climate warming [79], which means additional heat resources, with a positive effect on agricultural production season [80].

### 3.3. Step 3: Defining System Boundaries, Subsystems and Variables

**Figure 5:**
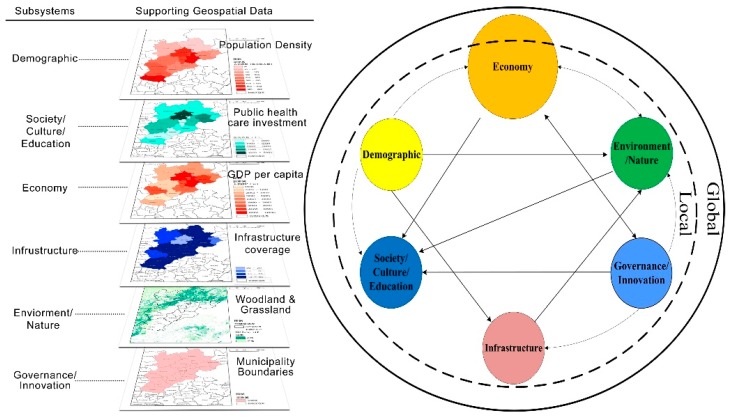
Illustration of six types of subsystems of the urbanization and eco-environment system. Source: Drawn by authors.

One way of understanding a complex system is to deconstruct it into components and define their boundaries. Given the complexity of the problem, we have to take cross-scale factors into consideration. For instance, at the global scale, FDI and trade of import and export can contribute to economic growth, generating increasing demand on land, water use and pollution risks. While at the local scale, population and economic growth, and investment in infrastructure will inevitably result in the increasing demand on water, land and energy use, and governance can aggravate or alleviate these influences. In this research, we divide the “Urbanization and Eco-environment system” into six types of subsystems, illustrated in Figure 5.

The “Urbanization and Eco-environment system” refers to a complex and dynamic system coupled by urbanization components of demographic, society, economy, infrastructure, governance and innovation, and eco-environment components of water, territorial, air, energy and creature in the context of rapid urbanization. (1) The demographic subsystem includes variables such as urban and rural population size, population density, population growth rate, immigration and emigration rate and urbanization rate; (2) In the society subsystem, variables such as employment/ unemployment, basic social security coverage rate, life expectancy, investment in public health care and education are identified; (3) The economy subsystem includes variables of foreign investment, total value of import and export trade, GDP, GDP per capita, investment in fixed assets and output values of primary industry, secondary industry and tertiary industry; (4) The infrastructure subsystem includes variables of highway mileage, traffic volume of various traffic systems, infrastructure coverage, mobile phone and network coverage; (5) The governance and innovation subsystem includes variables of government revenue and expenditure, environmental management capacity and investment in scientific and technological innovation; The eco-environment system includes variables of energy and resources, land use, biological environment, water environment and atmospheric environment. In addition, under the external influence of market environment, foreign investment labor support and climate change, the actual use of foreign capital, foreign direct investment, total import and export trade, population immigration/emigration rate, greenhouse gas concentration, agricultural production and average temperatures will be taken into index system (Table 3). 

### 3.4. Step 4: Defining the Interdependency of Components

The above-mentioned six subsystems and external influence factors connect to form an integrated “Urbanization and Eco-environment system.” In this model, there is a dynamic feedback relationship among different subsystems intermingled with external factors. In the context of globalization, the economy subsystem has significant influence on the employment/unemployment rate, government revenue and expenditure, innovation in science and technology, and population immigration and emigration. Various industries produce pollutants with a negative impact on environment. In the demographic subsystem, urban population size can not only affect economic scales, pollutants and traffic volume, but also generate higher demands on social security. In the infrastructure subsystem, pollution caused by transportation has an adverse influence on the atmospheric environment. In the governance and innovation subsystem, government expenditures play a dominant role in economy, ecological, society subsystem and infrastructure subsystems. While in ecological subsystem, policies of land and energy afford various opportunities for economic development.

**Figure 6:**
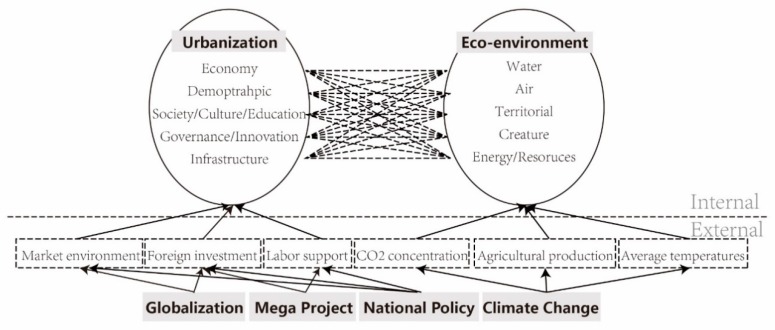
Internal correlation and external inflows of Urbanization and Eco-environment subsystems. Source: Edited according to Wang et al. [74].

Within a subsystem, there are a variety of causal feedback relationships among variables (Figure 6). Based on the feedback loop among the variables, causal relationships can be described as follows: (1) in the demographic subsystem, urban and rural population size is a key variable. Future population growth can be calculated according to the urbanization rate and population growth rate. After the urbanization level rises to some extent, the immigration and emigration rate of population declines and the urban population ceases growing; (2) In the society subsystem, an increasing investment in education can raise the employment rate, an increasing investment in public health care and expansion of basic social security coverage can extend life expectancy; (3) In the economy subsystem, FDI, total value of import and export trade, and investment in fixed assets not only influence output values of the primary, secondary and tertiary industries, but also contribute to changes in GDP and per capita GDP; (4) In the infrastructure subsystem, increasing infrastructure coverage can raise traffic volume and network coverage; (5) In the governance and innovation subsystem, government revenue and expenditures can exert direct or indirect impact on innovation in science and technology. In the eco-environment system, among land use factors, city growth not only gives rise to changes in various types of land use, but also leads to changes in biodiversity. Among water environment factors, all kinds of water use variables affect the sewage treatment variable resulting in water pollution. Among atmospheric environmental factors, various pollutant emission variables lead to changes in air quality.

In order to examine correlations among variables, we select one/two variables in each subsystem to conduct a bivariate analysis to test the interdependency between urbanization and eco-environment. We use population size, investment in education and health care, GDP, FDI, highway mileage, number of patents applied/granted, government revenue/expenditure, built-up area as surrogates of the demographic, social, economy, infrastructure and governance, and eco-environment subsystems and apply the panel data of the BTH region from 2000 to 2015. Table 4 reveals the results of the bivariate analysis, and all variables are significantly positively correlated.

Moreover, the urbanization and eco-environment system is a multi-level open and dynamic system, the internal development, external influence and eco-environment are interrelated, and mutually restrained. The complex relationships between them have both positive and negative influence on urban and eco-systems, and their coordination is a prerequisite for urban sustainable development. In order to identify the strength of coordination between the internal development level, external influence and eco-environment, the coupling coordination degree model in physics science was used in this paper. It could be used to characterize the degree of coordination between two or more systems [81], the calculation method could be seen in Equation (1):

$$C=3\times {\left\{\frac{I_{i}\times Env_{i}\times E_{i}}{{\left[I_{i}+Env_{i}+E_{i}\right]}^{3}}\right\}}^{\frac{1}{3}}$$

$$T=0.3I_{i}+0.4Env_{i}+0.3E_{i}$$

$$D=\sqrt{C\times T}$$

(1) $$D=\sqrt{3\times {\left\{\frac{I_{i}\times Env_{i}\times E_{i}}{{\left[I_{i}+Env_{i}+E_{i}\right]}^{3}}\right\}}^{\frac{1}{3}}\times \left(0.3I_{i}+0.4Env_{i}+0.3E_{i}\right)}$$

where *i* is the spatial area; *I_i_* is the internal development score of *i* region, *Env_i_* is the eco-environment score of *i* region, and *E_i_* is the external influence score of *i* region, all of them are evaluated with the weighted sum method by using normalized indicators mentioned above. D indicates the degree of coupling. D can be divided into four intervals: (1) D < 0.3, unbalanced development; (2) 0.3 < D < 0.6, barely balanced development; (3) 0.6 < D < 0.8, favourably balanced development; (4) D > 0.8 the, superiorly balanced [81,82,83].

**Figure 7:**
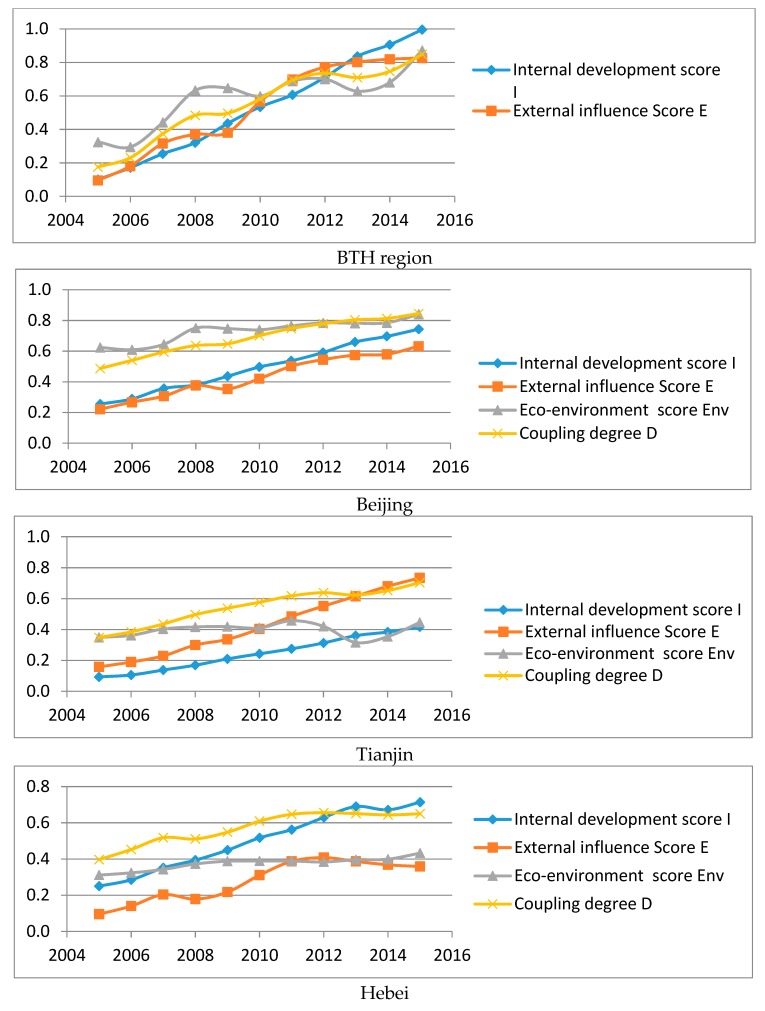
Coupling coordination degree of internal development, external influence, and eco-environment in BTH from 2005–2015. Source: Drawn by the authors.

Figure 7 reveals the result of coupling degree in BTH and Beijing, Tianjin and Hebei, respectively. As illustrated, the degree of coupling coordination (D) increased from seriously unbalanced development to barely balanced and then superiorly balanced development in BTH region in 2015, although there were some fluctuations. For Beijing, D kept growing and reached at superiorly balanced development in 2015; for Tianjin, a downturn of the eco-environment subsystem occurred in 2012, resulting in the decrease of coupling coordination degree; for Hebei, the degree of coupling coordination continued growing and the whole system reached a status of favourably balanced development after 2012.

### 3.5. Step 5: Run the Simulation Model to Assess and Predict the Impact of Urbanization on Eco-Environment

While analyzing the complicated and interactive relationship between multiple components, models such as Artificial Neural Network (ANN) and System Dynamics (SD) have been adopted [72,84]. In the case of BTH region, we take System Dynamics (SD) model as an example to evaluate interactions between urbanization and eco-environment. SD is an approach to understanding nonlinear behavior of complex systems over time, and a computer-aided approach to policy analysis and design. SD begins with defining problems dynamically, proceeds to steps for building confidence in the model and its policy implications. The basis of SD model is that there are many circular, interlocking, and sometimes time-delayed relationships among the components of urbanization and eco-environment system, which shape the behaviors of individual components. SD has been widely applied in many areas, and it is particularly helpful in research of interdependent population, economic, and ecological systems.

**Figure 8:**
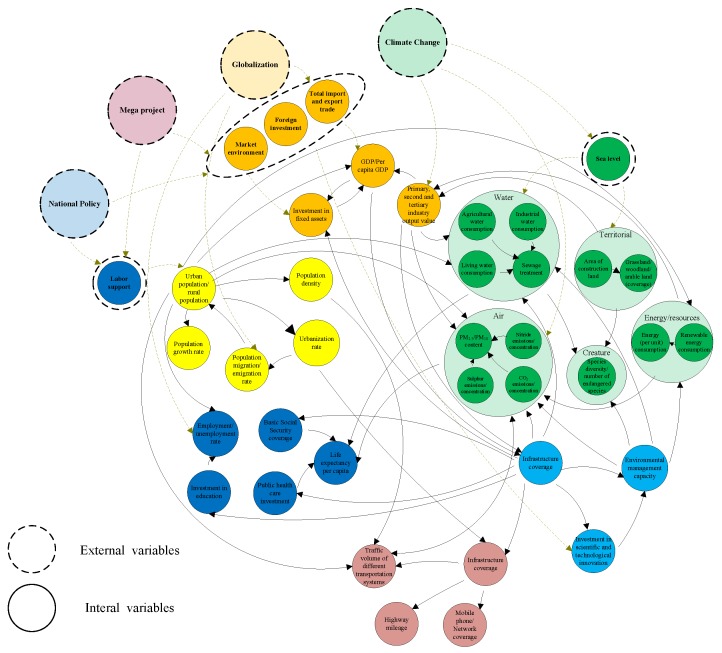
Stock and flow diagram of the Urbanization and eco-environment system in the BTH region.

Based on the above-mentioned interdependency evaluation among different subsystems and variables, a stock-flow diagram can be drawn, and then the SD model is constructed according to the characteristics and laws of subsystems (Figure 8). After the model is completed, it can be a systematic scientific platform for quantitative research of urbanization and eco-environment interaction. The platform can serve the following functions: (1) A basic simulation function: quantitative simulation of urbanization and eco-environment interaction in the dynamic urbanization process, which can reveal the evolution of the mechanism and laws between them; (2) An early warning function: the signal model can be used to alert for possible problems during the urbanization process and analyze problems; (3) A multi-scene analysis function: a number of scenarios can be designed in the process of long-term urbanization and ecological environment simulation, in order to help governments with their decision making process, for example, the impact of fertility and economic policies on energy use, the impact of transportation policies on pollutant emissions can be predicted [72]; (4) An external interface function: supporting the latest external data and allowing experts, researchers, and the public to use the model remotely to help in decision making.

## 4. Conclusions 

Rapid urbanization is a global phenomenon that has caused significant changes in many ecosystems, and generated ecological risks [47]. Untangling the relationship between urbanization and the eco-environment is complex and requires a multidisciplinary approach. Spatially, urbanization has heterogeneous impacts on ecosystems due to the disparity of urban expansion and population mobility in different places. In China, the rapid industrialization and urbanization process has resulted in dramatic urban growth and pollution, and growing demand for resources, increasingly challenging the long term sustainability of city-regions [85]. By taking the BTH region as an example, this research proposes a preliminary framework of analyzing mega city-regions through integrating the internal urbanization, external influence and eco-environment. The objective is to show how we can use this framework to systematically consider different components affecting eco-environment, and understand possible causal relationships, and potential outcomes of government policies and their consequence.

This conceptual framework can serve as a reference and guidance for policy-making in mega city-regions. It can provide a preliminary guide to the development of new approaches to understand and diagnose the casual relationship between urban policies and ecological consequences, and synthesize the complex components of mega city-regions in databases, and predict the potential impact of urban policies on the ecological system. Therefore, the proposed framework can contribute to possible solutions of sustainability problems of mega city-regions in a rapidly urbanizing context. Further empirical research applying this framework in different spatial scales is needed to test and optimize this model.

## Figures and Tables

**Table 1:** Existing frameworks of analysing urban and ecological systems.

	Theory	Components	Purpose	References	Application
City as a special type of ecosystem	Urban biodiversity & ecosystem	forests, grasslands, and wetlands	Assess the effect of urbanization on ecological system	Hamer & McDonnell, 2008; Wei et al., 2014 [10,38]	Urban forest and landscape management
	Urban metabolism	production, transformation, consumption and exchange of materials, resources, energy and services	Measure urban or regional socio-economic metabolism	Barles, 2010 [16]	Material and energy cycle assessment; Environmental footprint analysis
	Ecosystem services	provisioning services; regulating services; cultural services; supporting services	Provide ecological, environmental, economic, social and cultural benefits for mankind	Jones et al., 2013 [39]	Ecosystem service markets planning; Assessment of urbanization impacts
City as a coupled system	Ecological Economics	Four-Capital Framework	Balanced Eco-system of (1) built or manufactured capital; (2) human capital; (3) social capital and natural capital	Vemuri, 2006 [40]	Urban and regional planning approach
		Ultimate ends—Intermediate means—Ultimate means	Create an overarching goal with clear metrics of progress toward sustainable development	Costanza & Kubiszewski, 2016; Sustainable Development Goals (SDGs) (UN, 2014) [13,17]	Sustainable wellbeing model and measurement
	SES	Four core subsystems: resource systems, resource units, governance systems, and users	Organize different concepts and languages to describe and explain complex social-ecological systems (SESs)	Ostrom (2007, 2009); Grimm et al., 2008 [11,12,18]	Urban environmental stewardship; Urban-ecological network structure; Analysis of dynamics within urban SES
	DPSIR	Driver, pressure, state, impact, response	Develop an improved understanding of, indicators for, and appropriate responses to impact of human activities on the environment	Manap, 2012 [41]	Capture socioeconomic influential factors; Integration of ecosystem services and human well-being; DPSIR indicator system
City as a nexus system	Nexus Model	State, Market, Societal, and Geospatial	Bridging the State, Market, Societal, and Geospatial contexts	Jacobs, 2013 [42]	Complex urban-ecological system analysis; Governance promotion
	Smart city domains	Natural resources and energy, Transport and mobility, Buildings, Government, Economy and people	Nexus for sustainable development with daily life of human beings	Neirotti et al., 2014 [43]	Smart cities Designing, planning, and management; urban community transition; Innovation and governance promotion
	E-LAUD framework	Ecology—Environment & Human Health-Urban design management	Better understanding the complementary roles of ecological system in urban development and the functioning of ecosystems and ecological resilience in a complex human-dominated landscape	Kattel, 2013 [14]	Ecology-Environment and Human Health-Urban design management

**Table 2:** Social-economic development of BTH. (BHT: Beijing-Tianjin-Hebei.).

Region	Beijing			Tianjin			Hebei		
Year	2005	2010	2015	2005	2010	2015	2005	2010	2015
Population (million persons)	15.3	19.62	21.71	10.43	12.99	15.47	68.51	71.94	74.25
Urbanization rate	83.6%	86.0%	86.5%	75.1%	79.6%	82.6%	37.7%	44.5%	51.3%
GDP (billion yuan)	696.95	1411.36	2301.46	390.56	922.45	1653.82	1001.21	2039.43	2980.61
GDP per capita (yuan)	45,993	73,856	106,497	37,796	72,994	107,995	14,659	28,668	40,255
Industrial structure (Primary industry: secondary industry: tertiary industry) (%)	1.2:28.9:69.9	0.8:23.6:75.6	0.6:19.7:79.7	2.9:54.7:42.4	1.6:52.5:45.9	1.3:46.6:52.1	14:52.7:33.3	12.6:52.5:34.9	11.5:48.3:40.2
Annual disposable income of urban households (yuan)	17,563.0	29,073.0	52,859.0	12,638.6	24,292.6	34,101.0	9107.1	16,263.4	26,152.2
Annual disposable income of rural households (yuan)	7860	13,262	20,569	7202	11,801	18,482	3481.6	5958	11,050.5
Number of primary school students per teacher	10.3	13.2	14.3	13.0	13.6	15.0	15.6	16.0	18.6
Medical beds of per 1000 population	6.65	6.83	7.76	3.98	3.76	4.12	2.37	3.47	4.61

**Table 3:** Urbanization, eco-environment subsystems and variables.

		Subsystem/Inflows	Indicator
Internal variables	Urbanization	Demographic	Urban/rural population
			population growth rate
			population density
		Society	Employment/unemployment rate
			Life expectancy per capita
			Basic social security coverage
			Investment in education
			Investment in public health care
		Economy	GDP
			GDP of primary, second and tertiary industry
			Urbanization Rate
		Infrastructure	Highway mileageInfrastructure coverage
			Mobile phone/Network coverage
			Traffic volume of different transportation systems
		Governance/Innovation	Investment in science and technology innovation
			Government revenue/expenditure
	Eco-environment	Air	Sulphur Emissions
			Nitride emission
			PM_2-5_/PM_10_ content
		Creature	Species diversity/number of endangered species
		Territorial	Area of construction land
			Grassland/woodland/arable land (coverage)
		Energy/Resources	Renewable energy consumptionEnergy (per unit) consumption
		Water	Living water consumption
			Industrial water consumption
			Agricultural water consumption
			Sewage treatment
External flow		Globalization Mega Project National Policy Climate Change	Actual use of foreign capital
			Foreign direct investment
			Total import and export trade
			Population immigration/emigration rate
			Agricultural production
			Average temperatures
			Greenhouse gas concentration
		Subsystem/inflows	Indicator
Internal variables	Urbanization	Demographic	Urban /rural population
			population growth rate
			population density
		Society	Employment/unemployment rate
			Life expectancy per capita
			Basic social security coverage
			Investment in education
			Investment in public health care
		Economy	GDP
			GDP of primary, second and tertiary industry
			Urbanization Rate
		Infrastructure	Highway mileage Infrastructure coverage
			Mobile phone/Network coverage
			Traffic volume of different transportation systems
		Governance/Innovation	Investment in science and technology innovation
			Government revenue/expenditure
	Eco-environment	Air	Sulphur Emissions
			Nitride emission
			PM2-5/PM10 content
		Creature	Species diversity/number of endangered species
		Territorial	Area of construction land
			Grassland/woodland/arable land (coverage)
		Energy/Resources	Renewable energy consumption Energy (per unit) consumption
		Water	Living water consumption
			Industrial water consumption
			Agricultural water consumption
			Sewage treatment
External flow		Globalization Mega Project National Policy Climate Change	Actual use of foreign capital
			Foreign direct investment
			Total import and export trade
			Population immigration/emigration rate
			Agricultural production
			Average temperatures
			Greenhouse gas concentration

**Table 4:** Correlation test of urbanization and eco-environment indicators of BTH in 2000–2015.

	X_1_	X_2_	X_3_	X_4_	X_5_	X_6_	X_7_	X_8_	X_9_	X_10_	X_11_
X_1_	1										
X_2_	0.896 **	1									
X_3_	0.851 **	0.984 **	1								
X_4_	0.901 **	0.997 **	0.990 **	1							
X_5_	0.860 **	0.987 **	0.997 **	0.993 **	1						
X_6_	0.846 **	0.978 **	0.998 **	0.985 **	0.996 **	1					
X_7_	0.876 **	0.993 **	0.996 **	0.996 **	0.996 **	0.992 **	1				
X_8_	0.903 **	0.997 **	0.988 **	0.997 **	0.989 **	0.985 **	0.994 **	1			
X_9_	0.812 **	0.946 **	0.985 **	0.962 **	0.981 **	0.990 **	0.973 **	0.957 **	1		
X_10_	0.779 **	0.930 **	0.977 **	0.944 **	0.969 **	0.985 **	0.959 **	0.943 **	0.995 **	1	
X_11_	0.823 **	0.929 **	0.962 **	0.943 **	0.993 **	0.976 **	0.958 **	0.937 **	0.993 **	0.848 **	1

Note: X_1_ = Built-up Area; X_2_ = Population; X_3_ = Expenditure of Education & Medical and Health Care; X_4_ = GDP; X_5_ = Government Revenue; X_6_ = Government Expenditure; X_7_ = FDI; X_8_ = Highway Mileage; X_9_ = Number of patents applied; X_10_ = Number of patents granted; X_11_ = Actual use of foreign capital. ** Correlation is significant at the 0.01 level (2-tailed).
